# A pixel-wise annotated dataset of small overlooked indoor objects for semantic segmentation applications.

**DOI:** 10.1016/j.dib.2022.107791

**Published:** 2022-01-05

**Authors:** Elhassan Mohamed, Konstantinos Sirlantzis, Gareth Howells

**Affiliations:** School of Engineering, University of Kent, Canterbury, UK

**Keywords:** Semantic segmentation, Indoor objects, Door handles, Image dataset, Deep learning, Pixels classification, Convolutional neural network

## Abstract

The purpose of the dataset is to provide annotated images for pixel classification tasks with application to powered wheelchair users. As some of the widely available datasets contain only general objects, we introduced this dataset to cover the missing pieces, which can be considered as application-specific objects. However, these objects of interest are not only important for powered wheelchair users but also for indoor navigation and environmental understanding in general. For example, indoor assistive and service robots need to comprehend their surroundings to ease navigation and interaction with different size objects. The proposed dataset is recorded using a camera installed on a powered wheelchair. The camera is installed beneath the joystick so that it can have a clear vision with no obstructions from the user's body or legs. The powered wheelchair is then driven through the corridors of the indoor environment, and a one-minute video is recorded. The collected video is annotated on the pixel level for semantic segmentation (pixel classification) tasks. Pixels of different objects are annotated using MATLAB software. The dataset has various object sizes (small, medium, and large), which can explain the variation of the pixel's distribution in the dataset. Usually, Deep Convolutional Neural Networks (DCNNs) that perform well on large-size objects fail to produce accurate results on small-size objects. Whereas training a DCNN on a multi-size objects dataset can build more robust systems. Although the recorded objects are vital for many applications, we have included more images of different kinds of door handles with different angles, orientations, and illuminations as they are rare in the publicly available datasets. The proposed dataset has 1549 images and covers nine different classes. We used the dataset to train and test a semantic segmentation system that can aid and guide visually impaired users by providing visual cues. The dataset is made publicly available at this link.

## Specifications Table

**Table:** 

Subject	Computer science: Artificial Intelligence
Specific subject area	The provided dataset is annotated on the pixel level. Consequently, it is suitable for semantic segmentation (pixel classification) tasks. (Semantic segmentation, Computer vision, Deep learning, Object detection, Image processing, Convolutional neural networks)
Type of data	Images MATLAB images datastore MATLAB pixels datastore
How data were acquired	A one-minute video is collected while driving the powered wheelchair through the indoor environment corridors. The used camera to record the video is installed beneath the joystick of the powered wheelchair (the height of the camera from the ground is 68 cm). Data is then processed using MATLAB software (Video Labeller) to annotate video frames on the pixel level. The output of this process is two folders with the images and the corresponding annotations.
Data format	Original images (.png) Annotated images (pixel label data) (.png) Imds (images datastore) (.m) Pxds (pixels datastore) (.m)
Parameters for data collection	We have recorded a high-resolution video using the Intel® RealSense™ camera so that small objects, such as door handles, can comprise many pixels. This can enhance the features extraction process; consequently, accurate systems can be attained. The camera position, beneath the joystick, is chosen for the following reasons: the camera is integrated into the powered wheelchair body. There is no obstruction between the camera and the user's body. Lastly, the camera has a comparative perspective to the user field of view.
Description of data collection	The collected dataset has two folders for the images and the annotated pixels. Also, we included the two MATLAB files for image and pixel datastores which can be loaded in MATLAB software. Please note that the source file paths in images and pixels datastores should be modified to point to the new location of images and pixel labels folders.
Data source location	Institution: School of Engineering and Digital Arts, University of Kent City/Town/Region: Canterbury, Kent Country: UK Latitude and longitude (and GPS coordinates, if possible) for collected samples/data: 51.2984° N, 1.0640° E
Data accessibility	Repository name: Mendeley Data Data identification number: Mohamed, Elhassan (2021), “Indoor Semantic Segmentation Dataset”, Mendeley Data, V1, doi:10.17632/hs5w7xfzdk.1 Direct URL to data: https://data.mendeley.com/datasets/hs5w7xfzdk/1
Related research article	E. Mohamed, K. Sirlantzis and G. Howells, "Indoor/Outdoor Semantic Segmentation Using Deep Learning for Visually Impaired Wheelchair Users," in IEEE Access, vol. 9, pp. 147914-147932, 2021, doi:10.1109/ACCESS.2021.3123952.

## Value of the Data

• The categories covered by the proposed dataset are infrequent. Though, they are essential for many applications such as scene understanding and object manipulation.
• The provided dataset can help researchers in the computer vision and robotics communities to produce more robust systems that can segment and interact with multi-sized objects.
• Human-machine interaction applications can benefit from such a dataset as the covered classes, such as door handles, are essential for these applications.
• The proposed multi-purpose dataset is annotated at the pixel level for semantic segmentation tasks with high-resolution images and various object sizes.
• The dataset images can be easily loaded and used in many frameworks for experiments and trials using the accompanying Matlab datastore files.

## 1 Data Description

**Fig. 1:**
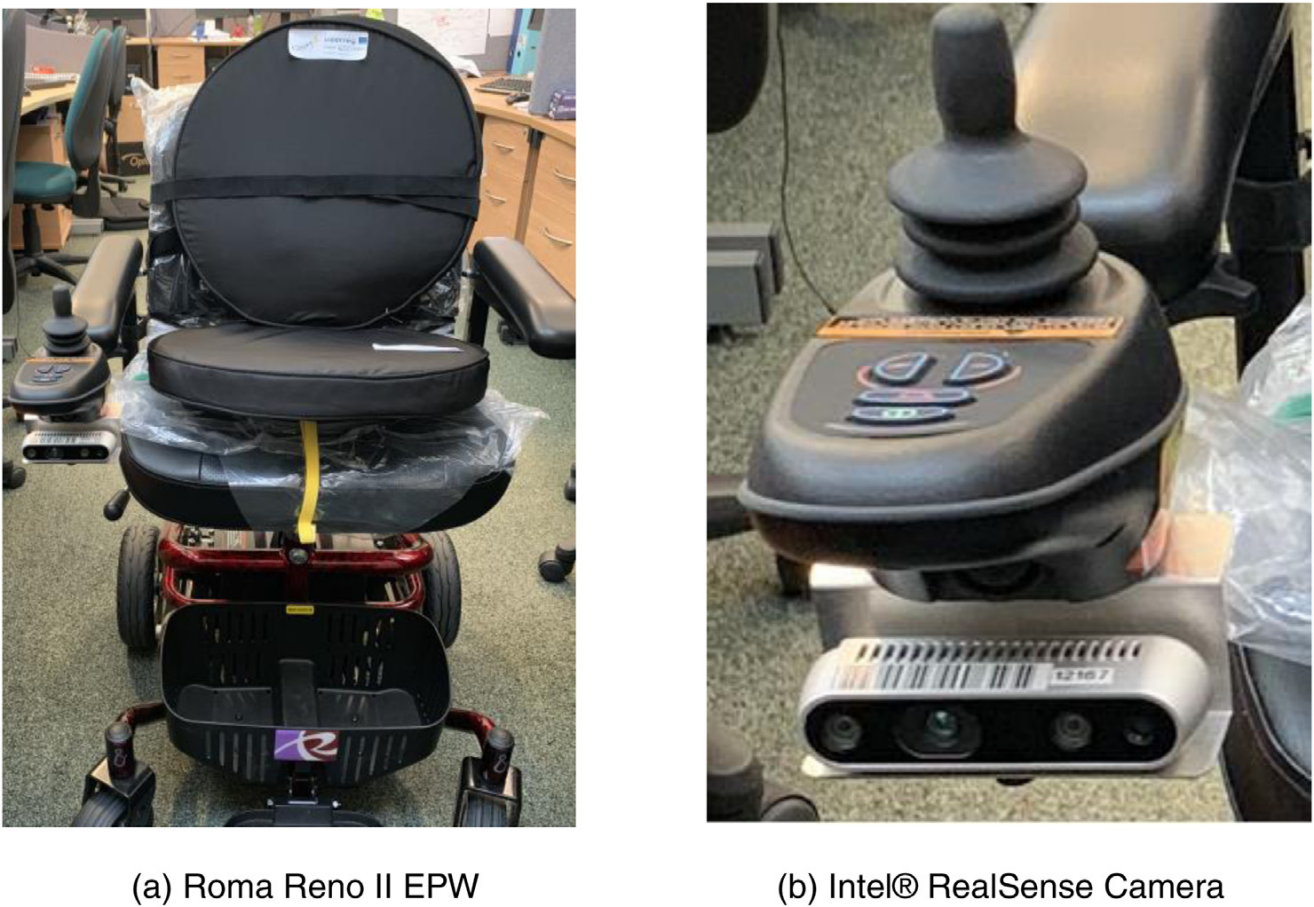
Camera installation for data collection.

**Fig. 2:**
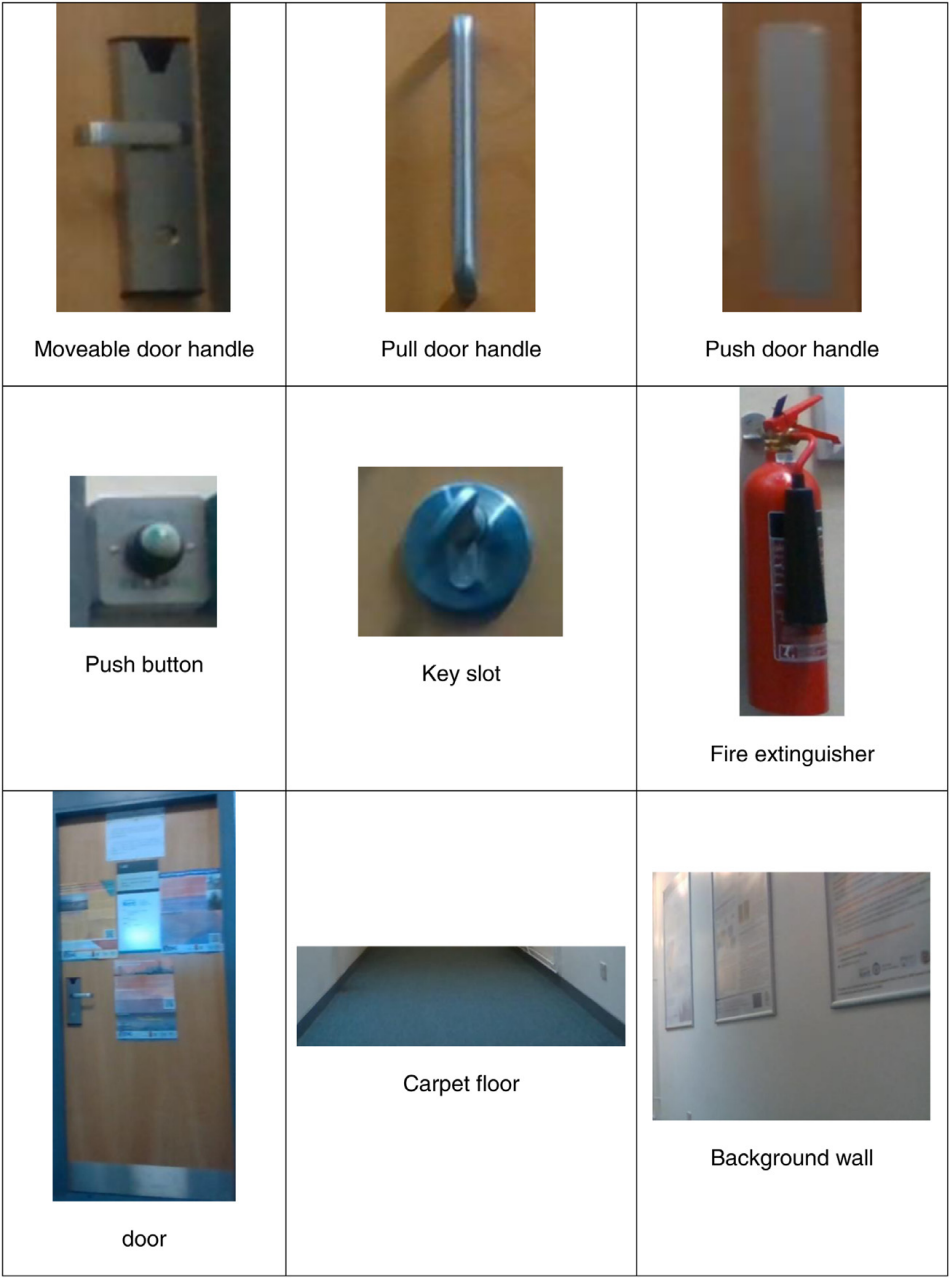
Indoor classes of interest of the proposed dataset.

The proposed dataset is introduced to fill the gap of lacking project-specific indoor objects of interest that a user may need to interact with on a daily basis (our project targets powered wheelchair disabled users). The system setup that has been used to collect the dataset is shown in Fig. 1. We focus on objects that can represent visual cues for visually impaired users or objects that disabled users may need to approach for further manipulation. These object categories are doors, floors, background walls, fire extinguishers, key slots, switches, and different kinds of door handles (push, pull, and moveable door handles). Fig. 2 shows the classes of interest of the proposed dataset. There are some publicly available datasets such as ADE20K [1,2] and SceneNN [3]. However, these datasets do not cover infrequent objects such as different kinds of door handles.

**Fig. 3:**
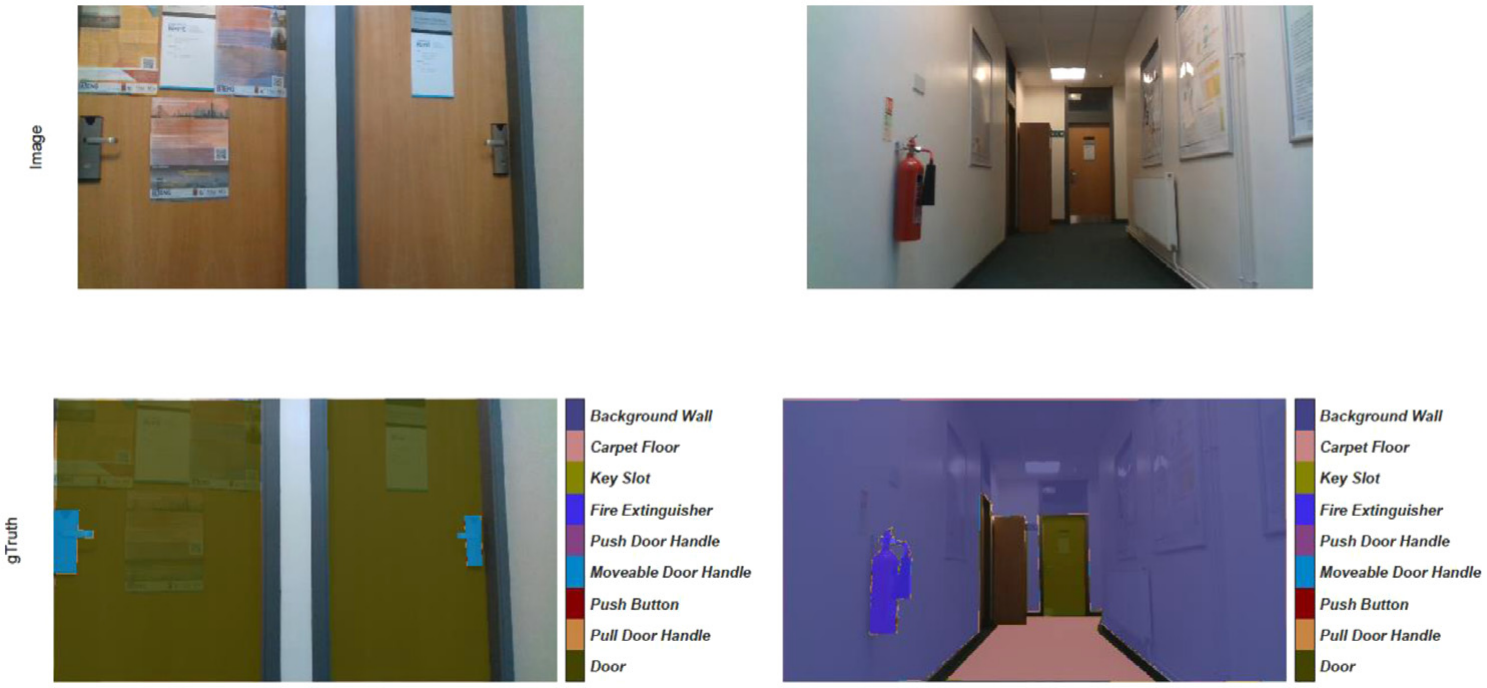
Examples from the collected dataset with the first row representing the images and the second row representing the corresponding pixels annotations.

Images extracted from the one-minute video are annotated manually by the first author and verified by the second. The dataset has 1549 images with image sizes of 960 × 540 × 3. Examples of the collected data with the ground truth annotation are shown in Fig. 3. Pixels that do not fit in any of the eight predefined classes are assigned to the Background wall class. However, small areas between two different classes, such as door frames, are kept unannotated. These pixels cannot be fit in the Background wall class as they belong to a different category of objects.

The proposed dataset images might look homogeneous as they have been collected from one trajectory. However, the collected objects are captured with different angles, orientations and light conditions. This makes the captured objects diverse, which can enhance the ability of the trained system to generalise to other scenarios. Data augmentation such as image rotation and scaling can be employed to overcome any potential limitations during training. Although the dataset can be used individually, it can also be combined with other datasets to enhance the objects' diversity and increase the number of objects instances.

**Fig. 4:**
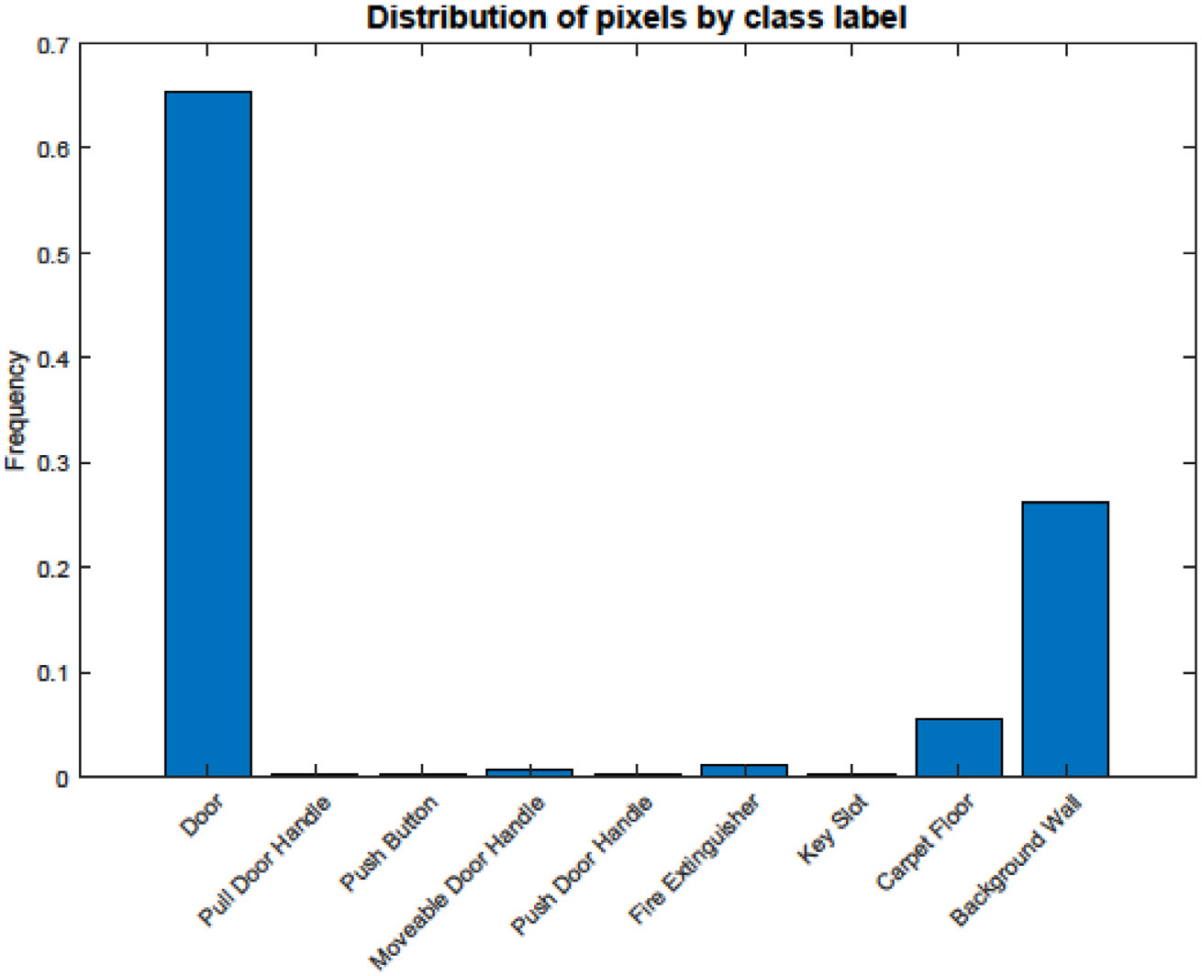
Pixels distribution in the proposed dataset.

It can be noticed from Fig. 4 and Table 1 that categories such as Doors and Background walls dominate the distribution of the pixels. In contrast, door handles have fewer pixels. This can be attributed to the objects’ sizes. Doors and Background walls represent the largest objects in the dataset compared to the other classes, which can be considered as small size objects. Though, the dataset has many object instances of all classes (Table 1).

**Table 1:** The number of annotated pixels per class and the number of object instances.

Class	Pixel count (Million)	Number of instances
Door	239.87	1742
Pull door handle	0.95	173
Push button	0.63	159
Moveable door handle	2.87	1134
Push door handle	0.78	262
Fire extinguisher	4.25	486
Key slot	0.78	216
Carpet floor	20.32	698
Background wall	96.40	398

## 2 Experimental Design, Materials and Methods

The Intel® RealSense Camera (Fig. 1b) is installed on the powered wheelchair (Fig. 1a) to capture a one-minute video while driving the powered wheelchair through the school corridors. This environment is selected because it represents a typical indoor environment of a work building or a corridor/lobby of a typical apartment. The video is then loaded into MATLAB video labeler software for pixel-level annotation. The camera operates at approximately 25 frames per second ‘FPS’. After annotation, we have exported the video to MATLAB software for further processing.

The exporting process converts the one-minute video into 1549 images with the corresponding pixels labeled images. All images have a size of 1920 × 1080 × 3. The dataset was then resized to 960 × 540 × 3. No further processing or normalisation is applied to the dataset.

In our experiments, we have done some preprocessing to the dataset before training, such as rescaling pixels values. However, we published the dataset without any normalisation to give the developers and researchers more flexibility to decide whether they need to apply any specific preprocessing techniques.

Splitting the dataset into train, validate, and test sets are left to the developers and researchers. However, we recommend two different splitting techniques. The first one is the random shuffling of the images and then split into the aforementioned sections. The second splitting technique is the hard split at which the first portion of the dataset is used for training, the second portion is used for validation, and the remaining portion is used for testing.

We have a very high pixels distribution of the Background walls and the Door classes, so we deliberately ignore annotating these categories in some frames. We believe this may help to balance the distribution of the pixels. Nevertheless, in our experiments, we applied frequency weightings to balance the classes weightings of the low-representative classes. We encourage this approach to avoid bias in favour of dominant classes.

## Ethics Statements

The authors generally followed the expected standards of ethical behaviour in scientific publishing throughout the article construction. However, the work did not involve the use or participation of human subjects or animals.

## CRediT authorship contribution statement

**Elhassan Mohamed:** Conceptualization, Methodology, Visualization, Formal analysis, Writing – original draft, Writing – review & editing, Data curation. **Konstantinos Sirlantzis:** Methodology, Validation, Resources, Supervision, Project administration, Writing – review & editing. **Gareth Howells:** Writing – review & editing, Supervision, Methodology.

## Declaration of Competing Interest

The authors declare that they have no known competing financial interests or personal relationships that could have appeared to influence the work reported in this paper.
